# Strategies for Preventing Nosocomial Influenza in Acute-Care Hospitals: A Narrative Review

**DOI:** 10.3390/medicina62020344

**Published:** 2026-02-09

**Authors:** Wei-Hsuan Huang, Yi-Fang Ho, Jia-Jen Jang, Hsien-Po Huang, Ting-Kuang Yeh, Chia-Wei Liu, Chien-Hao Tseng, Yan-Chiao Mao, Chun-Mei Ho, Jheng-Yi Yeh, Yu-Fen Chen, Yu-Yueh Shih, Pei-Chun Pan, Chun-Hsi Tai, Yu-Hsia Hen, Hsin-Yi Hung, Pei-Hsuan Huang, Po-Yu Liu, Po-Hsiu Huang

**Affiliations:** 1 Division of Infectious Diseases, Department of Internal Medicine, Taichung Veterans General Hospital, Taichung 407, Taiwan; 2 Infection Control Center, Taichung Veterans General Hospital, Taichung 407, Taiwan; 3 Genomic Center for Infectious Diseases, Taichung Veterans General Hospital, Taichung 407, Taiwan; 4 School of Medicine, College of Medicine, National Yang Ming Chiao Tung University, Taipei 112, Taiwan; 5 Graduate Institute of Biomedical Engineering, National Chung Hsing University, Taichung 402, Taiwan; 6 Graduate Institute of Molecular Biology, National Chung Hsing University, Taichung 402, Taiwan; 7 Department of Medical Toxicology, Taichung Veterans General Hospital, Taichung 404, Taiwan; 8 School of Medicine, National Defense Medical Center, Taipei 114, Taiwan; 9 Department of Post-Baccalaureate Medicine, College of Medicine, National Chung Hsing University, Taichung 402, Taiwan

**Keywords:** nosocomial influenza, healthcare personnel vaccination, infection control, patient safety, cross-infection

## Abstract

Seasonal influenza remains a major threat to healthcare facilities, where introduction of the virus can cause disproportionate morbidity and mortality among high-risk inpatients. This narrative review synthesizes current evidence and practice-oriented guidance on the prevention of hospital-acquired influenza. We conducted a targeted literature search using PubMed, guideline repositories for English-language publications from 2000 to 2025, prioritizing systematic reviews, clinical trials, and authoritative guidelines. A multifaceted strategy is emphasized: annual vaccination of healthcare personnel and eligible patients; consistent implementation of standard and transmission-based precautions; attention to environmental cleaning and disinfection; and occupational-health policies that limit presenteeism and workplace exposure. Evidence demonstrates that higher healthcare personnel (HCP) vaccination coverage is associated with lower patient influenza rates and improved survival. Reliable hand hygiene, respiratory source control, early initiation of droplet precautions, and cohorting when single rooms are limited all contribute to interrupting in-facility transmission. Ensuring that ill HCP can remain off duty without penalty further reduces the likelihood of staff-to-patient spread. Collectively, these coordinated measures provide a protective framework and underscore the central role of clinicians and infection-prevention teams in sustaining influenza control within acute-care settings.

## 1. Introduction

Seasonal influenza remains a substantial and recurrent threat to global public health and the safety of patients and healthcare personnel [1]. Annual epidemics infect up to 1 billion people worldwide, cause 3–5 million severe cases, and result in 290,000–650,000 influenza-associated respiratory deaths globally per year based on pre-pandemic modeling [1]. In the United States, recent seasons have produced marked interseasonal variability, historically causing 9–41 million infections and 6300–52,000 deaths annually [2]. Reflecting a return to significant seasonal activity, estimates for the 2023–2024 season indicate a burden at the higher end of this spectrum, with approximately 41 million illnesses and 25,000 deaths [3], illustrating the recurrent and substantial stress on acute-care services. A similar burden is evident in subtropical Asian regions like Taiwan. According to surveillance data from the Taiwan Centers for Disease Control [4], distinct from the strict winter seasonality in temperate zones, Taiwan often experiences year-round influenza circulation with secondary surges in the summer months. For hospitalized patients, often older, multimorbid, or immunocompromised, nosocomial acquisition is particularly consequential, being associated with higher morbidity, more than a twofold increase in length of stay, and substantially elevated in-hospital mortality compared with uninfected inpatients [5].

While many seasons are manageable within routine preparedness frameworks, the 2017–2018 U.S. season demonstrated that influenza could approach the upper end of expected severity, contributing to ≈52,000 deaths and sustaining pressure on healthcare delivery [2]. Superimposed on this seasonal burden is the enduring pandemic potential of influenza. While the recent COVID-19 pandemic vividly demonstrated the fragility of global healthcare systems against respiratory pathogens, historical precedents specific to influenza—such as the 1918 pandemic (≈50 million deaths) and the rapid global spread of 2009 H1N1—confirm that novel influenza viruses possess a similar capacity to overwhelm hospital response capacity [6]. This necessitates sustained vigilance beyond the immediate post-COVID-19 era.

Healthcare facilities can paradoxically act as amplification settings once influenza is introduced. Entry may occur via an incubating patient, an infectious visitor, or a symptomatic or presymptomatic healthcare worker (HCW); in environments of close patient–staff contact, shared equipment, and multi-bed rooms, spread can be rapid [7]. Risks are most significant in oncology, transplant, and hematology units, where profound immunosuppression makes nosocomial influenza especially morbid and sometimes fatal [8]. HCWs who work while infectious can drive bidirectional transmission between staff and vulnerable patients; without rapid institution of transmission-based precautions, a single case can precipitate a unit-based cluster [7,8].

Within this epidemiologic context, clinicians are central to prevention. High influenza vaccination coverage among HCWs is a cornerstone of patient safety, and clinicians contribute by being vaccinated themselves and by promoting vaccination among colleagues and patients [7]. Equally important is the reliable execution of everyday infection-prevention practices, which directly reduce opportunities for transmission [7]. Early recognition of suspected influenza and timely placement under droplet precautions enables rapid containment, while adherence to occupational health and sick leave policies provides essential source control [9].

This narrative review synthesizes guidance and the infection-prevention literature to outline facility-level strategies to mitigate influenza transmission, protect high-risk inpatients, and strengthen preparedness for seasonal and pandemic influenza [7,9].

## 2. Materials and Methods

We conducted a targeted narrative review to synthesize evidence relevant to the prevention and control of hospital-acquired influenza. A comprehensive literature search was performed across PubMed, Google Scholar, and the official Centers for Disease Control and Prevention (CDC) and World Health Organization (WHO) guideline repositories. Search terms included combinations of keywords using Boolean operators, such as (‘Influenza’ OR ‘Seasonal Influenza’) AND (‘Nosocomial’ OR ‘Hospital-acquired’ OR ‘Health-care associated’) AND (‘Prevention’ OR ‘Control’ OR ‘Vaccination’). Reference lists of key articles and guideline documents were also manually screened to identify additional relevant publications. Initial screening was performed by the primary author (W.-H.H.), with full-text inclusion decisions validated by the corresponding author (P.-H.H.) to ensure relevance to clinical practice.

Literature published between 2000 and 2025 was prioritized to capture both pre- and post-COVID-19 infection-control practices. We included systematic reviews, randomized or cluster-randomized trials, observational epidemiologic studies, outbreak investigations, implementation studies, and major public-health guidelines applicable to acute-care hospital settings. Commentaries, editorials, single-case reports, laboratory-only virology studies, and articles focused exclusively on community settings were excluded. Due to the substantial heterogeneity in study settings (acute care vs. long-term care), outcome definitions (laboratory-confirmed influenza vs. ILI), and vaccination ascertainment methods across the included literature, a formal meta-analysis was not conducted.

Data Synthesis and Visualization: While a quantitative meta-analysis was not performed, we extracted key outcome data (e.g., odds ratios and 95% confidence intervals) from selected high-quality studies to illustrate the range of reported effect sizes. 

## 3. Results

**Figure 1:**
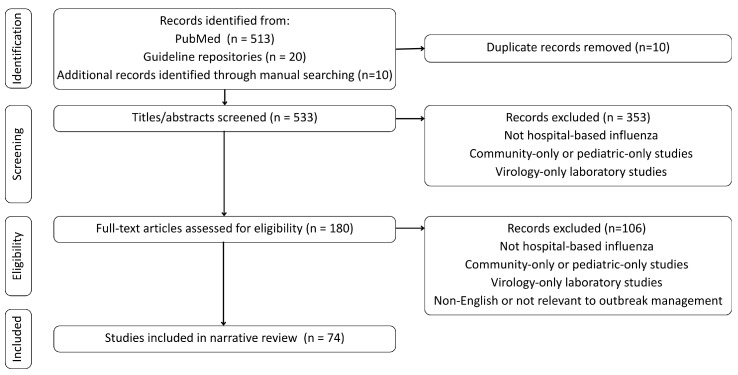
Flow diagram of study selection for the narrative review.

Across all databases and sources, the initial search retrieved 543 records. After removal of duplicates and screening of titles and abstracts for relevance to nosocomial influenza, infection-prevention strategies, or hospital-level influenza control, 180 articles underwent full-text review. Of these, 74 peer-reviewed publications and authoritative guideline documents were deemed to meet inclusion criteria based on clinical relevance, methodological rigor, applicability to hospital practice, and alignment with the scope of this review. Study selection for the narrative review is illustrated in Figure 1.

A total of 543 records were identified through database and guideline searches. After removing duplicates, 533 records underwent title and abstract screening. Of these, 353 were excluded primarily because they focused on community-based transmission, pediatric-only populations not applicable to general adult wards, or basic virology without clinical correlates. The remaining 180 full-text articles were assessed for eligibility. Ultimately, 74 studies met the inclusion criteria and were included in the final synthesis.

### 3.1. Epidemiology and Transmission

Effective prevention of healthcare-associated influenza depends on understanding how the virus spreads and which host or environmental conditions increase risk in hospitals [10]. Seasonal flu is transmitted mainly by respiratory droplets generated at close range (≈6 feet) during coughing, sneezing, or talking; this droplet-dominant pattern supports routine medical masking and droplet precautions in acute-care settings [10]. Transmission can also occur through direct contact with secretions or indirectly via contaminated surfaces, because the influenza virus can persist for minutes on skin and for 24–48 h on common nonporous materials, making hand hygiene and environmental cleaning essential [10]. Although long-range airborne spread is less common, aerosol-generating procedures (e.g., bronchoscopy, intubation, open suctioning) create respirable particles and warrant higher-level respiratory protection (N95) and appropriate engineering controls [11].

Rapid clinical recognition is equally important because it activates isolation. While the classic influenza syndrome is well described, hospitalized patients often present atypically: older adults may be afebrile with delirium or functional decline; immunocompromised patients may have blunted symptoms; children may have higher fevers or gastrointestinal complaints [10]. Co-circulation of SARS-CoV-2 (severe acute respiratory syndrome coronavirus 2) and RSV (Respiratory syncytial virus) further complicates bedside diagnosis. Thus, during periods of known community influenza activity, a low threshold for testing and empiric droplet precautions helps prevent unrecognized ward transmission [10].

Virologic features justify early, layered control. Incubation is short (1–4 days), infectiousness begins about 24 h before symptoms, peaks early, and typically lasts 5–7 days in healthy adults but can extend to 10 days in adults and to 2–3 weeks or longer in young children and severely immunocompromised patients [10]. Because presymptomatic shedding and prolonged infectivity are common in high-risk groups, symptom-based strategies alone are insufficient; vaccination, standard precautions, prompt isolation, and environmental hygiene must be used together [9].

The post-2020 period adds new challenges. During COVID-19 (Coronavirus disease 2019), broad nonpharmaceutical interventions were followed by a collapse in global influenza circulation, illustrating the power of coordinated source control [12]. After these measures were relaxed, several regions reported out-of-season activity, earlier peaks, and markedly higher influenza levels, up to a 715% increase in parts of South America, consistent with an “immunity gap” or “immunity debt” [13,14]. At the same time, childhood influenza vaccination in the United States declined by about five percentage points, and adult coverage did not rise enough to compensate, leaving more susceptible patients, visitors, and staff [15]. Consequently, healthcare facilities may now face faster or larger clusters, especially when influenza co-circulates with SARS-CoV-2 or RSV [13]. Current priorities are to maximize vaccination of healthcare personnel and eligible inpatients, maintain the ability to scale up source control, isolation, and cohorting based on surveillance, and enforce sick-leave and work-restriction policies to prevent staff-driven introduction [13]. In this altered landscape, the consistent use of evidence-based infection-prevention measures remains essential [10,11,12,13,14,15].

### 3.2. Healthcare Personnel Vaccination: A Primary Prevention Strategy

Annual influenza vaccination of healthcare personnel (HCP) is a cornerstone of hospital influenza-prevention programs and the most direct means of reducing staff-mediated introduction and transmission of influenza in clinical settings [16,17,18]. Because HCP have frequent, close, and sometimes prolonged contact with medically fragile patients, they can inadvertently act as sources of infection, even when illness is mild or unrecognized; in some seasons, up to 23% of HCP may be infected, and many of these infections are subclinical yet still potentially transmissible [16]. Vaccinating HCP protects individual workers and, more importantly, adds a protective layer for high-risk inpatients, older adults, immunocompromised people, and patients with multiple comorbidities, as well as for other staff who might otherwise be exposed in patient-care areas [17]. This interruption of staff-to-patient spread is critical, since hospital-acquired influenza outbreaks are well documented and associated with severe outcomes in high-risk units [17]. Accordingly, CDC’s HICPAC (Healthcare Infection Control Practices Advisory Committee) and ACIP (Advisory Committee on Immunization Practices) strongly recommend achieving and maintaining high influenza vaccination coverage among HCP, which also helps preserve workforce capacity during seasonal surges [17,18].

#### 3.2.1. Evidence for Patient Protection

**Figure 2:**
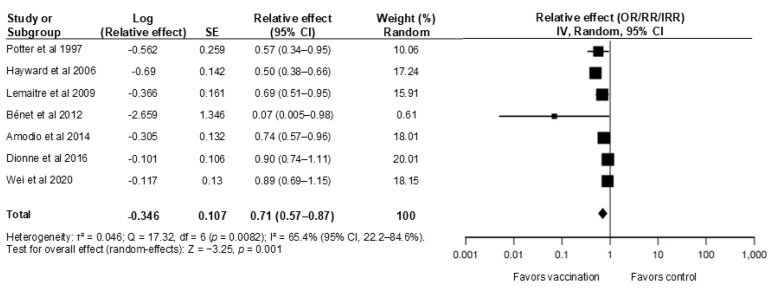
Pooled relative effect of healthcare-worker influenza vaccination on nosocomial influenza among hospitalized patients. Outcomes include laboratory-confirmed hospital-acquired influenza, and, for cluster-randomized trials, influenza-like illness as reported in the original studies [20,21,23,24,25,26,27]. Dionne 2016 [23], Amodio 2014 [24], and Wei 2020 [25]) express a +10% increase in HCW vaccination coverage; Bénet 2012 [27] compares ≥35% vs. <35% coverage. Abbreviations: OR, odds ratio; aOR, adjusted odds ratio; RR, risk ratio; IRR, incidence rate ratio; CI, confidence interval; SE, standard error; IV, inverse-variance; I^2^, heterogeneity (I-squared); τ^2^, between-study variance (tau-squared); df, degrees of freedom.

The protective effect of HCP vaccination on patient outcomes has been demonstrated across multiple care settings and study designs. Table 1 and Figure 2 summarize selected key studies demonstrating this impact. Facilities with higher HCP vaccination coverage consistently report lower rates of healthcare-associated influenza, fewer influenza-like illness (ILI) episodes among patients, and possible reductions in all-cause mortality [19,20,21].

A 2014 systematic review by Ahmed et al. observed that institutions with higher HCP vaccination coverage had significantly better patient outcomes, including a 29% reduction in all-cause mortality (pooled RR 0.71, 95% CI 0.59–0.85) and a 42% reduction in patient ILI (RR 0.58, 95% CI 0.46–0.73) compared with institutions with lower coverage [19]. These findings align with earlier randomized trials in long-term and geriatric care. In a landmark cluster-randomized trial, Potter et al. reported that vaccinating approximately 61% of staff in geriatric hospital units was associated with a 44% reduction in influenza-related mortality among patients (OR 0.56, 95% CI 0.40–0.80) and a 43% reduction in ILI (OR 0.57, 95% CI 0.34–0.94) [20]. Another cluster-randomized trial in long-term care homes found that increasing staff vaccination coverage from 5% to 35% was associated with fewer influenza and ILI cases, fewer hospitalizations, and ≈5 fewer deaths per 100 residents in intervention facilities compared with controls [21]. A pair-matched cluster-randomized trial in 40 nursing homes likewise found that higher staff vaccination coverage (69.9% vs. 31.8%) was associated with lower resident all-cause mortality and ILI and less staff sick leave. However, hospitalization rates were not significantly different [26]. Together, these findings support routine influenza vaccination of staff caring for institutionalized older adults.

Evidence from acute-care hospitals indicates that higher vaccination coverage among healthcare personnel can correlate with lower rates of hospital-acquired, laboratory-confirmed influenza [22,27,28]. Although some landmark randomized controlled trials (e.g., Potter et al., Hayward et al. [20,21]) were conducted in long-term care facilities, their findings provide essential biological proof of concept for herd immunity. These data remain highly relevant to acute-care settings, particularly for units with longer lengths of stay (e.g., geriatric, rehabilitation, or psychiatric wards), serving as a proxy where acute-care-specific RCTs are limited. Salgado et al. observed a significant inverse association after coverage rose from 4% to 67% (*p* < 0.001) [22]. Bénet and Amour observed a significant inverse association between HCP vaccination and nosocomial influenza. Specifically, Bénet et al. reported a substantial reduction in risk (adjusted OR 0.07, 95% CI 0.005–0.98) when vaccination coverage exceeded approximate thresholds, although the wide confidence intervals suggest variability in the magnitude of effect [27,28]. In an Italian acute-care hospital, a seven-season retrospective analysis (2005–2012) similarly found an inverse association between HCW vaccination coverage and nosocomial influenza-like illness, with each percentage-point increase in coverage associated with a small but statistically significant reduction in non-ILI risk (adjusted OR 0.97, 95% CI 0.94–0.99) [24].

However, Dionne et al. detected no significant change despite coverage increasing from 47% to 90%, likely reflecting strain variability or high baseline control measures [23]. Similarly, an ecological study from a Singapore tertiary hospital (2013–2018) found no statistically significant association between HCW influenza vaccination coverage and monthly nosocomial influenza incidence after adjustment for background influenza activity and testing intensity, although the point estimate (IRR 0.89, 95% CI 0.69–1.15) suggested a possible protective effect [25]. A Cochrane review likewise found limited impact on laboratory-confirmed influenza but noted reductions in ILI and related healthcare use [29].

#### 3.2.2. Voluntary Versus Mandatory Vaccination Policies

To realize these protective effects, healthcare organizations employ voluntary or mandatory approaches. Voluntary programs, education, convenient on-site access, modest incentives, improve uptake but often plateau at moderate coverage (≈40% in many institutions in the early 2000s; frequently <60% without explicit requirements) [18,30]. Mandatory policies, such as annual immunization requirements with medical exemptions and, where applicable, alternatives like continuous masking or employment consequences, achieve substantially higher coverage and have become more common. U.S. hospitals reporting mandatory HCP vaccination increased from about 37% (2013) to 61% (2017) [29]. During 2018–2019, overall HCP vaccination averaged 81% but was highest in settings where vaccination was required (97.7%) versus largely voluntary long-term care settings (≈67.9%) [30]. Coverage at or near these levels approaches commonly cited thresholds for adequate herd protection in healthcare (≈90–94%) and correlates with fewer nosocomial events, particularly in high-risk units [18].

#### 3.2.3. Occupational and Operational Benefits

Beyond patient protection, HCP vaccination supports workforce stability: vaccinated staff are less likely to acquire influenza, reducing sick leave during seasonal surges [18]. In an Italian hospital (2017–2018), unvaccinated HCP had higher illness-related absence than vaccinated HCP (2.47 vs. 1.92 lost workdays per 100 person-days; *p* < 0.001), ≈22% less absenteeism among vaccinated personnel [16]. In the UK National Health Service, improving staff vaccination coverage was associated with a ≈0.4-percentage-point reduction in staff sickness absence, corresponding to a 9% relative reduction from a 4.5% baseline [31]. These operational advantages reinforce HCP influenza vaccination as both a patient-safety intervention and a system-level strategy for sustaining safe staffing during influenza season [16,18,31].

### 3.3. Integrated Prevention Strategies

Effective prevention requires a bundled approach where vaccination and non-pharmaceutical interventions reinforce each other to break the chain of transmission.

#### 3.3.1. Vaccination of Healthcare Personnel and Patients

Hospitalized patients, particularly older adults, the immunocompromised, and those with chronic cardiopulmonary or metabolic disease, are the group most likely to experience severe influenza-related outcomes. About 90% of seasonal influenza–related deaths and more than half of influenza-associated hospitalizations occur in adults ≥ 65 years, underscoring the concentration of risk in this population [32]. Immunocompromised individuals, including solid-organ and hematopoietic stem cell transplant recipients and patients receiving cytotoxic or biologic therapies, are even more vulnerable: they may develop more severe disease, respond less predictably to antivirals, and shed viruses for prolonged periods, thereby increasing the likelihood of transmission in inpatient settings [33]. Nosocomial influenza in these groups is associated with poor outcomes; outbreak reports note median case-fatality rates of 16%, rising to 60% in highly immunosuppressed cohorts such as transplant recipients [34]. Modeling suggests that timely inpatient vaccination can prevent a meaningful proportion of hospital-acquired influenza in such high-risk populations [34].

Clinicians, therefore, occupy a critical position in operationalizing inpatient immunization. Best practice is to assess vaccination status at admission or early in the hospital stay, and to vaccinate all eligible, unvaccinated patients during the current influenza season [33]. This approach aligns with national quality metrics, including The Joint Commission’s IMM-2 measure, which emphasizes screening and vaccinating before discharge [35]. To improve uptake, many hospitals use standing orders or protocol-based pathways that authorize nursing or pharmacy personnel to administer vaccines without a separate provider order for every patient [36]. Embedding vaccine prompts in admission order sets and providing brief counseling further narrows immunity gaps, turning hospitalization into a “reachable moment” that reduces the pool of susceptible inpatients and, consequently, the risk of onward influenza transmission within the facility.

Multiple strategies have successfully increased inpatient influenza vaccination, with standing orders emerging as especially effective. When vaccination is built into admission, transfer, or discharge workflows, procedural barriers are minimized; one program reported an inpatient vaccination rate of 40.3%, substantially higher than in hospitals lacking such mechanisms [36]. In a randomized trial, computerized standing orders outperformed physician reminders (42% vs. 30%), indicating that automated, protocol-based approaches drive greater uptake than reminder-only strategies [37]. Subsequent reviews have consistently identified standing orders as among the most effective single interventions for adult inpatient immunization [38]. Embedding prompts, default vaccine orders, and eligibility checks into the electronic medical record (EMR) can further standardize practice; EMR-based programs have achieved vaccination rates of ≥80% by ensuring that every admission triggers vaccine consideration [39]. Provider education enhances these system changes: combining education with manual standing orders increased vaccination to 43%, greater than either alone, suggesting a synergistic effect between institutional and clinician-level interventions [40]. Reminder systems aimed at clinicians or patients provide reinforcement, though their independent impact is usually smaller than that of standing orders or EMR integration [37,40].

The largest, most durable improvements have come from multi-component programs that integrate standing orders, EMR prompts, education, audit-and-feedback, and visible administrative support, raising inpatient vaccination from about 60% to >80% and yielding a 74% relative increase in orders in one implementation study [39,41]. Table 2 summarizes selected interventions and their observed impact on inpatient influenza immunization.

#### 3.3.2. Non-Pharmaceutical Interventions (NPIs)

While vaccination provides the biological foundation of immunity, its limitations—such as variable effectiveness and coverage gaps—necessitate the concurrent application of non-pharmaceutical interventions (NPIs). These measures, including hand hygiene, standard precautions, and environmental disinfection, act synergistically with vaccination to create multiple barriers against transmission.

Hand hygiene is the most fundamental infection-prevention measure and the primary defense against healthcare-associated transmission of influenza and other respiratory pathogens [44]. The World Health Organization’s “My 5 Moments for Hand Hygiene” framework offers a behaviorally grounded model that directly targets droplet- and fomite-mediated spread in acute care [45]. Both soap-and-water handwashing and alcohol-based hand rubs (ABHR) effectively remove or inactivate the influenza virus; ABHR is preferred for routine care due to its rapid virucidal activity, better skin tolerability, and higher observed compliance [44]. Point-of-care availability of ABHR is strongly associated with sustained improvements and lower infection rates [44].

Meta-analytic data indicate that hand hygiene performed > 10 times daily can substantially reduce respiratory virus transmission, and multifaceted interventions have achieved 50–70% reductions in healthcare-associated infections [46,47]. However, compliance frequently remains < 50% and is hindered by workload, time pressure, and skin irritation; these barriers can be improved through product optimization, visible leadership modeling, and unit-level feedback [44,48,49]. Consistent hand hygiene by all care team members remains a simple, high-yield strategy to reduce the risk of nosocomial influenza [46].

Respiratory hygiene and cough etiquette are complementary source-control measures aimed at reducing the emission of infectious droplets at their point of origin [50,51]. Core elements include covering coughs and sneezes, proper tissue disposal, and immediate hand hygiene; facilities should supply tissues, masks, and ABHR in clinical and waiting areas and post clear signage to standardize expectations for patients and visitors [50,51].

Early identification and masking of individuals with acute respiratory symptoms is central to this approach. Surgical masks worn by symptomatic people substantially reduce the expulsion of virus-laden droplets and protect others more effectively than masks worn only by uninfected contacts [52,53]. Clinicians should also encourage mildly symptomatic HCP who remain at work—when permitted by occupational health policies—to wear a mask to protect patients and colleagues [11]. Although surgical masks do not eliminate aerosol transmission, they substantially reduce droplet spread, the principal mode for seasonal influenza, and thus support other preventive measures [52].

Droplet precautions are the primary transmission-based measure for limiting influenza spread in healthcare facilities. They should be instituted promptly for any patient with suspected or confirmed infection, without awaiting laboratory confirmation, particularly when community activity is high [51,54]. Key elements include single-room placement or cohorting with other influenza cases, use of a surgical mask for personnel on room entry, eye protection when splash or spray is likely, and masking of patients during transport with advance notice to the receiving service [51,54]. Precautions are generally maintained for 7 days after symptom onset or 24 h after fever resolution, with longer durations for immunocompromised hosts who may shed virus longer [51].

When rigorously implemented, nosocomial influenza can be substantially reduced; a structured ward-based protocol (DroPS) was noninferior to single-room isolation, indicating that adherence can offset limited isolation capacity [54]. Aerosol-generating procedures require the use of N95 respirators and, when feasible, airborne isolation, and consistent application is associated with fewer and shorter outbreaks [54,55].

### 3.4. Facility and Policy Measures

Beyond direct patient care, facility-level interventions create the operational environment necessary for effective infection prevention. Both environmental hygiene and sick-leave policies represent critical system-level controls; however, their real-world effectiveness is frequently compromised by similar structural barriers, including high workload, staffing shortages, and institutional culture. Addressing these compliance gaps requires shifting focus from individual vigilance to robust administrative support.

#### 3.4.1. Environmental Cleaning: Clinician Role in Surface Hygiene

Hospital surfaces can serve as critical transient reservoirs for influenza virus, making environmental hygiene a core element of outbreak prevention and control in acute-care settings [56]. Infected patients shed virus-laden droplets into their immediate surroundings, and experimental data show that influenza A and B can remain viable for 24–48 h on hard, nonporous surfaces (e.g., plastic, bed rails) and for several hours on porous materials such as fabrics [56]. Because a viable virus can be readily transferred from contaminated surfaces to the hands of healthcare personnel or other patients, inadequate or omitted hand hygiene creates a direct pathway for fomite-mediated transmission [57]. Accordingly, thorough and frequent cleaning followed by disinfection of high-touch and patient-proximal surfaces is essential. Standard protocols, mechanical removal of soil, and application of an Enduring Power of Attorney (EPA)-approved hospital disinfectant for the manufacturer’s stated contact time are generally sufficient because influenza is an enveloped virus and relatively easy to inactivate [58].

Although environmental services personnel perform scheduled room cleaning, clinicians have an indispensable, complementary role in disinfecting shared, non-critical patient-care equipment that moves between rooms, such as stethoscopes, blood pressure cuffs, oximeter probes, portable ultrasound devices, because such items can facilitate cross-infection if not disinfected between uses [59]. Guidance recommends wiping these devices with an appropriate hospital-grade disinfectant, following manufacturer instructions and institutional infection-prevention policies, and ensuring adequate wet contact time (often ≈1 min) to achieve virucidal effect [59,60]. Alcohol-based wipes are well-suited for small surfaces and high-frequency workflows [61]. Clinicians should also decontaminate any visibly soiled surface in their immediate care area without waiting for routine cleaning rounds.

A broad range of agents rapidly inactivate the influenza virus. Laboratory studies confirm the effectiveness of 70% ethanol or 1-propanol, often within one minute [62]; standard dilute sodium hypochlorite solutions (≈1%) remain highly active, even with moderate organic load [63]; and quaternary ammonium compounds, especially when combined with alcohol, offer a practical option for daily hospital use [64]. For more extensive decontamination, hydrogen peroxide vapor has been employed, while several novel formulations, such as CAC-717 and iron-based disinfectants, have shown promising virucidal activity in experimental work [65,66]. Additional technologies under evaluation include photosensitizer-coated antiviral wipes and conventional antiseptic molecules such as cetylpyridinium chloride (CPC), though activity may vary by concentration and matrix [67,68]. Importantly, many of these agents retain efficacy in the presence of organic material, which is critical in real-world clinical environments [63,69].

The pivotal requirement for frontline staff is not access to exotic products but consistent, protocol-driven use of EPA-approved disinfectants on all high-touch surfaces and shared devices, thereby lowering environmental viral burden and reducing opportunities for indirect transmission [57]. Nevertheless, observed compliance is frequently suboptimal, audits repeatedly show low rates of stethoscope disinfection among physicians, indicating a gap between policy and practice [70]. Sustained improvement therefore depends on ongoing education, visible leadership modeling, point-of-care product availability, and embedding surface-disinfection steps into routine clinical workflows. When combined with hand hygiene, source control, and transmission-based precautions, disciplined environmental cleaning substantially mitigates the risk of influenza propagation within hospitals. Table 3 summarizes selected disinfectants and their demonstrated activity against influenza virus based on current literature [56,57,58,59,60,61,62,63,64,65,66,67,68,69,70].

#### 3.4.2. Sick-Leave Policies: Preventing Healthcare Worker-Mediated Transmission

HCW represents a direct threat to patient and staff safety because symptomatic clinicians can become unrecognized sources of influenza transmission within healthcare facilities. Viral shedding can persist for several days and may continue as symptoms improve, permitting silent spread in high-contact clinical areas [71,72]. Multiple outbreak investigations have traced nosocomial clusters to a single contagious HCW, highlighting the need for clear, operational sick-leave policies embedded in infection-prevention programs. Presenteeism, working while ill, has been shown to contribute materially to intrahospital transmission of influenza and other respiratory viruses, especially when masking, hand hygiene, or isolation practices are inconsistently applied or poorly modeled by senior staff [73,74]. Allowing symptomatic personnel to remain at home reduces immediate transmission risk and helps maintain workforce continuity by preventing secondary infections among coworkers and patients.

Despite guidance to stay off duty with influenza-like illness, HCWs often experience pressure to work, professional norms, concern about staffing, reluctance to burden colleagues, and institutional cultures that prioritize attendance over safety [72,75]. These pressures intensify during peak influenza activity or staffing shortages, when HCW-to-patient transmission is most consequential. Evidence shows that proactive, explicitly supported sick-leave policies, particularly those that guarantee paid leave and are endorsed by leadership, promote early symptom reporting and reduce on-duty work during infectious periods [43,76]. In one hospital, paid sick leave was associated with a significant decline in staff influenza-like illness (*p* < 0.01) and fewer HCW-linked clusters. Accordingly, prompt self-reporting and exclusion from duty until clinical clearance (e.g., afebrile ≥ 24 h without antipyretics) should be regarded as core infection-control requirements, supported by visible leadership and reliable paid leave [55].

## 4. Discussion

### 4.1. The Necessity of a Multimodal Strategy

Prevention of nosocomial influenza relies on the deliberate, coordinated, and consistently executed use of multiple complementary strategies, as outlined in the preceding sections. The available evidence shows that no single intervention affords sufficient protection in the complex, high-contact environment of acute-care facilities; maximal effect is achieved only when measures are implemented as an integrated bundle that acts at several points along the transmission pathway.

Vaccination of HCP is the logical first line of defense because it reduces the likelihood that influenza will be introduced or propagated by staff who frequently interact with patients at heightened risk [17,19,20]. Parallel promotion and administration of influenza vaccination for inpatients provides direct protection to those most vulnerable to severe outcomes, including older adults, immunocompromised hosts, and individuals with substantial comorbidities [32,34].

These immunization elements are strengthened by routine adherence to standard and transmission-based precautions. Consistent, moment-appropriate hand hygiene interrupts contact- and fomite-mediated transmission routes [44,46,49]; respiratory hygiene and source control reduce droplet dispersion at its origin [51,52]; and prompt initiation of droplet precautions for patients with suspected influenza establishes an immediate barrier around the infectious source [54,77]. Concurrent, meticulous environmental cleaning and disinfection of shared equipment and high-touch surfaces decrease the contribution of the inanimate environment as a transient reservoir for influenza virus [56,57,58,59]. Finally, occupational-health policies that support exclusion from work while symptomatic are indispensable to prevent infectious HCP from serving as unrecognized transmission sources [55,71,74].

The rationale for such layering and redundancy is compelling. Each measure addresses a distinct potential point of failure in the chain of transmission, and each has known limitations. Even in settings with high vaccine uptake, breakthrough infections occur and viral shedding may persist; in these instances, rigorous hand hygiene, respiratory etiquette, and the correct use of PPE serve as secondary or tertiary safeguards [44,51,52]. Likewise, environmental decontamination effectively reduces surface bioburden but cannot prevent droplet spread during close clinical encounters if masking and droplet precautions are neglected [54,56]. A multilayered approach, therefore, acknowledges the partial effectiveness of individual interventions while maximizing overall protection by multiplying opportunities to interrupt transmission. This redundancy principle, long recognized in other high-reliability sectors, is increasingly accepted in healthcare as fundamental to patient safety.

Within this framework, the actions of individual clinicians remain pivotal. Institutional policies, supplies, and protocols create an enabling environment, but day-to-day professional conduct determines whether these measures translate into fewer hospital-acquired infections. Clinicians who receive annual influenza vaccination, facilitate vaccination for eligible patients, perform hand hygiene reliably, use PPE as indicated, model respiratory hygiene, verify disinfection of shared equipment, and stay home when ill measurably enhance patient and coworker protection [17,44,51,56]. Visible adherence by senior staff and unit leaders can normalize these behaviors and foster unit-level accountability; nonetheless, individual commitment must be sustained by system-level support.

Accordingly, healthcare facilities bear responsibility for removing structural barriers to adherence. Ready access to vaccines for staff and inpatients, reliable availability of appropriate PPE, and placement of alcohol-based hand rub at the point of care are practical prerequisites for consistent practice [44,45]. Clear, non-punitive sick-leave policies that genuinely allow symptomatic HCP to remain off duty are essential to limiting staff-to-patient and staff-to-staff transmission [55,71,74]. Ongoing education, competency-based training, and periodic reinforcement help maintain awareness of current guidance and consolidate expected behaviors. In parallel, robust facility-level surveillance for influenza, informed by community activity, enables timely escalations such as intensified masking, visitor restrictions, or cohorting when signals of increased transmission arise. Embedding these elements into routine workflow, supported by explicit institutional commitment and adequate resources, is central to minimizing the clinical and operational impact of influenza in hospitals.

### 4.2. Challenges in Implementation

However, translating these evidence-based strategies into practice faces significant real-world limitations. First, vaccine effectiveness (VE) varies substantially by season and strain match; during years of significant antigenic drift, VE can drop below 40%, meaning that even high vaccination coverage may not fully prevent outbreaks. This inherent variability underscores why vaccination must be paired with physical barriers rather than relied upon as a standalone shield. Second, adherence to infection prevention measures is often hindered by systemic barriers. Hand hygiene compliance frequently plateaus below 50% due to workload and skin irritation [44]. Similarly, despite clear sick-leave policies, healthcare personnel often continue to work while symptomatic (“presenteeism”) due to staffing shortages and professional norms that prioritize continuity of care [72,75]. Acknowledging these hurdles is essential for designing resilient infection-control systems.

### 4.3. Future Directions

Looking ahead, several technological and biomedical advancements promise to reshape hospital influenza prevention. First, next-generation vaccines, including mRNA platforms and universal influenza vaccine candidates, aim to broaden protection across strains and reduce the impact of antigenic drift. Second, the integration of artificial intelligence (AI) into hospital surveillance systems could enable real-time prediction of nosocomial clusters by analyzing EMR data, allowing for preemptive rather than reactive isolation. Finally, engineering controls such as continuous active disinfection technologies (e.g., far-UV light) offer a passive defense layer that reduces reliance on human behavioral compliance for environmental hygiene.

### 4.4. Limitations of This Review

Finally, this review has several limitations. First, as a narrative review, the selection of literature was purposive rather than exhaustive. Unlike a systematic review, we did not employ dual-blind screening or formal quality assessment tools (e.g., GRADE, ROBINS-I) to evaluate the risk of bias for each included study. This methodology allows for a broader synthesis of diverse evidence sources but may introduce selection bias. Second, due to the substantial heterogeneity in study settings (acute vs. long-term care) and outcome measures across the included literature, we did not perform a quantitative meta-analysis. Third, our recommendations rely heavily on guidelines from high-resource settings (e.g., U.S. CDC, Taiwan) and English-language publications, which may limit generalizability to resource-constrained environments or non-English speaking regions.

## 5. Conclusions

The clinician’s role in preventing seasonal and pandemic influenza in acute-care settings is central to the success of institutional infection-prevention programs. Hospitals concentrate on vulnerable hosts and promote close-contact care, allowing the introduced influenza virus to be amplified and transmitted to patients with limited physiologic reserve [7,8]. Hospital-acquired influenza in such populations is associated with excess morbidity, prolonged hospitalization, and higher mortality, underscoring the need for rigorous frontline prevention [3,5].

Effective control is necessarily multi-layered and depends on clinicians consistently applying evidence-based measures. Annual influenza vaccination of healthcare personnel, together with active facilitation of vaccination for eligible inpatients and staff, remains the most effective preemptive intervention. Although evidence suggests variability in specific outcomes, high vaccination coverage is a fundamental pillar of patient safety and a central component of best practice within a multimodal infection-control strategy [17,19,32,34,35]. Routine use of standard and transmission-based precautions—including meticulous hand hygiene [44,49], respiratory hygiene and source control (rapid masking of symptomatic individuals) [51,52], and prompt initiation of droplet precautions for suspected influenza [51,52]—is equally indispensable. Clinicians further limit indirect transmission by ensuring appropriate disinfection of shared equipment and high-touch surfaces [56,58,59]. Finally, adherence to occupational-health and sick-leave policies, mainly exclusion from work while symptomatic, is a critical form of source control that protects patients and coworkers [43,55,71,75].

## Figures and Tables

**Table 1:** Summary of Key Representative Studies on Healthcare Worker Influenza Vaccination and Patient Outcomes in Acute-Care and Long-Term Care Settings.

Study (Year, Setting)	HCP Vaccination Rate	Patient Outcome & Measure	Outcome Result (Effect Size)
Ahmed et al. 2014 (Systematic review in hospitals & LTC facilities) [19]	Varied (multi-study analysis)	Patient all-cause mortality (pooled)	↓ Mortality by 29% in higher HCP coverage settings (RR 0.71, 95% CI 0.59–0.85); also ↓ ILI by 42% (RR 0.58)
Potter et al. 1997 (Cluster RCT in LTC geriatric hospitals) [20]	~61% of staff vaccinated	Influenza-related mortality in residents	↓ Mortality by 44% in intervention units (OR 0.56, 95% CI 0.40–0.80); significant reduction in resident ILI (OR 0.57).
Hayward et al. 2017 (Cluster RCT in long-term care facilities) [21]	Increased from 5% to 35%	Influenza cases, ILI, and complications in residents	Fewer influenza cases and ILI in vaccinated-staff homes; noted ~5 fewer deaths per 100 residents vs. controls, with reduced hospitalizations and GP visits
Salgado et al. 2004 (Tertiary-care hospital, USA) [22]	Increased from 4% to 67%	Nosocomial (hospital-acquired) influenza cases	Significant decline in nosocomial influenza post-vaccination campaign (*p* < 0.001)
Dionne et al. 2016 (Academic medical center, Canada) [23]	Increased from 47% to 90%	Nosocomial influenza incidence	No significant change detected despite high coverage (OR 0.99, 95% CI ~0.97–1.01; *p* > 0.1)

Key and abbreviation: ↓ denotes a reduction in outcome incidence; HCP = healthcare professional; OR = odds ratio; RR = risk ratio; ILI = influenza-like illness; LTC = long-term care; GP = general practitioner. Note: Due to the large number of included records (*n* = 74), this table presents a summary of representative studies that highlight critical outcomes in both acute-care and long-term care settings. It is not an exhaustive list of all literature analyzed in this review.

**Table 2:** Selected studies of inpatient influenza vaccination interventions and outcomes.

Study (Year)	Intervention(s)	Outcome: Inpatient Vaccination Rate
Cohen et al. (2015) [39]	Multi-component program (hospital-wide campaign, embedded EMR vaccine orders, daily reminders, standing orders)	Increased from ~60% to >80% of patients vaccinated (≈33% relative increase)
Rao et al. (2019) [41]	Multi-component program (web-based staff education, reminders, EHR prompts/lists, financial incentives)	Vaccine order rate increased from 28.8% to 50.2% (74% relative increase)
Dexter et al. (2004) [37]	Computerized standing orders (automated EMR order system) vs. physician reminders	42% of eligible inpatients vaccinated with standing orders, vs. 30% with provider reminders.
Donato et al. (2007) [40]	Standing order policy + provider education intervention	43% of inpatients vaccinated (the highest rate among interventions tested in that study)
Crouse et al. (1994) [36]	Standing orders protocol (nurse-driven vaccination offer to all eligible patients)	40.3% of inpatients vaccinated under the program (95.2% of patients were offered the vaccine)
Lawson et al. (2000) [42]	Standing orders protocol (tertiary care hospital)	22% higher inpatient vaccination rate compared to the surrounding community’s rate (due to the hospital program)

Sources: Adapted from a narrative review of inpatient influenza vaccination program effectiveness [32,38,43] and individual study results as cited above. These studies collectively demonstrate that systematized interventions (especially standing orders and EMR-integrated approaches, often in combination with education and reminders) consistently lead to substantial improvements in influenza vaccination rates among hospitalized patients. Key and abbreviation: EMR = electronic medical recor.

**Table 3:** Summary of selected disinfectants effective against the influenza virus on surfaces, with typical concentrations, contact times, and outcomes.

Disinfectant	Concentration	Contact Time	Influenza Inactivation
Ethanol (ethyl alcohol)	~70% (*v*/*v*)	1 min	Complete inactivation (no viable virus) [62]
1-Propanol (n-propanol)	~70% (*v*/*v*)	1 min	Complete inactivation (no viable virus) [62]
Sodium hypochlorite (bleach)	~1% (active chlorine)	~1 min (<60 min)	Complete inactivation of virus [63] (≥99.99% kill)
Solvent/detergent mix (Triton X-100 + TnBP)	1.0% + 0.3%	1 min	Complete inactivation (no detectable virus) [62]
Quaternary ammonium + alcohol (e.g., disinfectant wipe)	(product-specific)	Not reported	Eliminated live virus on surfaces [64]
CAC-717 (novel agent)	~1120 mg/L	1 min	>3 log_10_ reduction (>99.9% inactivation) [65]
Iron-based formulation	–	10 min	≥6 log_10_ reduction (≥99.9999% inactivation) [66]
Photosensitizer-coated wipe	–	N/A (dry wipe)	~5 log_10_ reduction in viral titer [67]
Cetylpyridinium chloride	~10–250 μg/mL (tested range)	5–90 min	~2 log_10_ reduction at ≈20 μg/mL [68]

Key and abbreviation: TnBP = tri(n-butyl) phosphate; *v*/*v* = volume of solute over volume of total solution. Complete inactivation indicates no infectious virus detectable in assays, while log_10_ reduction values denote the factor by which the viral titer decreases. N/A, not applicable (contact time not applicable for dry wipe–based disinfection).

## Data Availability

No new data were created or analyzed in this study. Data sharing is not applicable to this article.

## Abbreviations

The following abbreviations are used in this manuscript:

HCW	healthcare worker
CDC	Centers for Disease Control and Prevention
WHO	World Health Organization
SARS-CoV-2	Severe acute respiratory syndrome coronavirus 2
RSV	Respiratory syncytial virus
COVID-19	Coronavirus disease 2019
HCP	healthcare personnel
ILI	influenza-like illness
CPC	cetylpyridinium chloride
